# Systematic bioinformatic analysis of expression levels of 17,330 human genes across 9,783 samples from 175 types of healthy and pathological tissues

**DOI:** 10.1186/gb-2008-9-9-r139

**Published:** 2008-09-19

**Authors:** Sami Kilpinen, Reija Autio, Kalle Ojala, Kristiina Iljin, Elmar Bucher, Henri Sara, Tommi Pisto, Matti Saarela, Rolf I Skotheim, Mari Björkman, John-Patrick Mpindi, Saija Haapa-Paananen, Paula Vainio, Henrik Edgren, Maija Wolf, Jaakko Astola, Matthias Nees, Sampsa Hautaniemi, Olli Kallioniemi

**Affiliations:** 1 Medical Biotechnology, VTT Technical Research Centre and University of Turku, Itäinen pitkäkatu 4C, Turku, Finland; 2 Institute for Molecular Medicine Finland (FIMM), University of Helsinki, Tukholmankatu 8, Helsinki, Finland; 3 Department of Signal Processing, Tampere University of Technology, Korkeakoulunkatu 1, Tampere, Finland; 4 Department of Cancer Prevention, Institute for Cancer Research, Rikshospitalet-Radiumhospitalet Medical Centre, Oslo, NO-0310, Norway; 5 Computational Systems Biology Laboratory, Institute of Biomedicine and Genome-Scale Biology Research Program, University of Helsinki, Haartmaninkatu 8, Finland

## Abstract

Our knowledge on tissue- and disease-specific functions of human genes is rather limited and highly context-specific. Here, we have developed a method for the comparison of mRNA expression levels of most human genes across 9,783 Affymetrix gene expression array experiments representing 43 normal human tissue types, 68 cancer types, and 64 other diseases. This database of gene expression patterns in normal human tissues and pathological conditions covers 113 million datapoints and is available from the GeneSapiens website.

## Abstract 2

A method for the comparison of mRNA expression levels of most human genes across gene expression array experiments, and a database of the results, are presented.

## Background

A fundamental challenge in the post-genome era is the identification of the context-specific functions of human genes across healthy and disease tissues. Thousands of gene expression microarray measurements are performed each year by the scientific community and many of the data are made publicly available. In order to make use of this resource, integration of large collections of gene expression data from different tissues and microarray platforms is required. Available datasets, however, are often discordant and challenging to integrate due to the variety of the technologies used. Nevertheless, meta-analyses have already been shown to facilitate the analysis of gene expression across healthy and disease states [1-3]. Due to the use of various microarray platforms in studies, the multiple datasets are typically analyzed separately [4-9], for instance, focusing on cancer-normal comparisons within an organ type. Other studies have looked for systematic co-expression patterns between genes across multiple datasets in order to predict functions of genes [1,3,10-15]. While this is useful for the understanding of common shared functions of genes across different organs, highly tissue- or disease-specific gene functions may be missed.

Here, we describe the development of a database of *in silico *transcriptomics data that currently integrates 157 separate studies involving 9,783 human specimens, from 43 normal tissue types, 68 cancer types and 64 other disease types. The launch of the database was made possible by the development and validation of a novel method to normalize data arising from different Affymetrix microarray generations. The array data are linked with detailed clinical classifications and endpoints and are available through an interactive web interface designed for exploration by biologists and available at the GeneSapiens website [16]. We demonstrate here the application of the GeneSapiens system to the tissue- and disease-specific expression profiles of human genes one at a time or as gene clusters.

## Results and discussion

### Overview of the *in silico *transcriptomics data in the GeneSapiens system

The database was constructed from 9,783 CEL files of Affymetrix based gene expression measurements from normal and pathological human *in vivo *tissues and cells. We selected data from the five most widely used Affymetrix array generations (HG-U95A, HG-U95Av2, HG-U133A, HG-U133B, HG-U133 Plus 2), which were then normalized together. The detailed contents of the database are described in Additional data files 3 and 4. Each sample was systematically manually annotated with detailed information (when available) on sample collection procedures, demographic data, anatomic location, disease type, and clinicopathological details. These integrated data make it possible to generate expression profiles of any gene across 175 human tissue and disease types.

Custom software was developed to construct the database from the collection of CEL files and manually curated annotations linked to each sample. The software was based upon a Perl wrapper calling several subprograms written in Perl, R [17], C++ and MySQL and Linux Bash scripts. The subprograms identify unique CEL files by using cyclic redundancy checks, preprocess the files, perform the normalization steps, fetch gene annotations from Ensembl and incorporate the manually made annotation for each sample, create a complete MySQL database and perform the final integrity checks. Visualization and analysis tools were implemented in R [17], and the processed data are made available through a user-friendly and interactive web site [16]. We also implemented a virtual machine approach, the final result being a hardware-independent and rapidly installable complete operating system optimized for running the GeneSapiens database and web-server for the visualization interface.

### Development of the data normalization procedure

We implemented a three-step normalization strategy that consisted of probe-level preprocessing, equalization transformation (Q) and array-generation-based gene centering (AGC). We demonstrate that these steps resulted in data that are comparable across the major Affymetrix array generations.

#### Step I: data preprocessing at the probe level

We first used the MAS5.0 method [18] to preprocess raw data in the .CEL files. MAS5.0 is an optimal algorithm for the purpose of analyzing very large datasets [19] as it requires less memory than other widely used methods, and the biological representativity of the MAS5.0 normalized data is well documented [19]. In the three-step normalization approach, the subsequent normalization stages also minimized possible problems generated by the MAS5.0 preprocessing algorithm.

Importantly, we mapped the probes from each array generation type directly to Ensembl gene IDs by using alternative CDF files (version 10) [20] to avoid inaccuracies generated by the original probeset design of Affymetrix arrays. Therefore, this resulted in the optimal redefinition of the gene specificities of the probes and excluded those probes that, according to the recent genome assembly, mapped to multiple genes or nowhere in the genome.

#### Step II: Q normalization

After preprocessing, we performed sample-wise normalization of the entire dataset at the gene level. This was done by equalization transformation [21] (Q), which is similar to the widely used quantile normalization [22] in which the samples are transformed by substituting their values with the means of quantiles in the entire dataset. In the Q procedure, we transformed each sample to follow a normal distribution that was estimated from the log_2_-transformed values of the entire dataset (Additional data file 1). The estimated parameters were a mean of 8 and standard deviation of 2. This step of the sample-wise normalization was necessary to prevent a small number of aberrant samples from dominating the mean values for genes within an array generation used in the AGC correction.

#### Step III: array-generation-based gene centering (AGC normalization)

We developed a novel AGC method to avoid the bias caused by the different oligos quantifying the same gene in the different Affymetrix array generations. The AGC method is based on the availability of data, on each array generation, from a large number of samples representing different tissues or diseases. In the AGC method, a correction factor is calculated for each gene in each array generation. These correction factors are then used to normalize the gene expression distributions across the whole database (see Materials and methods for details).

### Validating the entire normalization protocol

**Figure 1:**
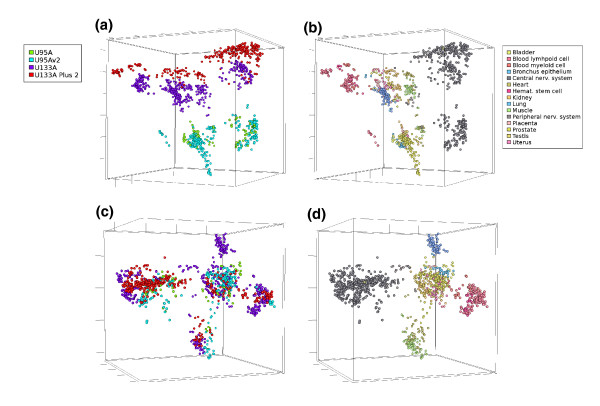
Multidimensional scaling (MDS) of Q normalized data before and after AGC correction. MDS was performed using 1,137 healthy *in vivo *samples representing 15 tissue categories with 7,390 genes in common without missing values. Color codes show the array generation of each sample for panles on the left-hand side and the high level anatomical system from which samples originate for panels on the right-hand side. **(a, b) **Clustering of samples in Q normalized data without AGC correction. (a) Clustering driven dominantly by the array generations, but some biological division can be seen in the form of some division within the large clusters. (b) Several tissue classes are separated into two or more clusters due to the different array generation of origin. **(c, d) **After QAGC, array generations no longer define clusters (c) but instead tissue types form distinct clusters (d).

**Figure 2:**
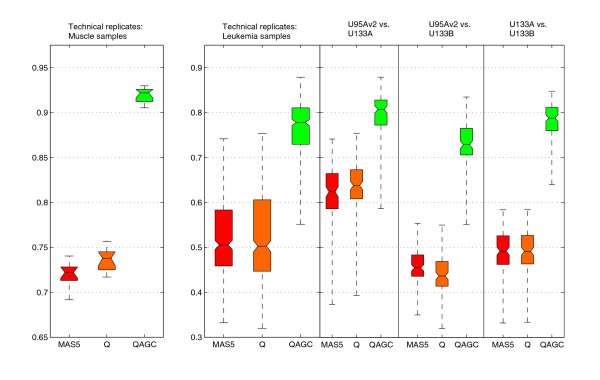
Boxplots of correlations between the replicated samples after each step of the data normalization process. All boxes for which notches do not overlap vertically have significantly (α = 0.05) different median values. On the left is a sample set from 14 human muscle biopsy samples measured with array generations U95Av2 and U133A. The correlations computed based on the QAGC-normalized data are significantly higher when compared to MAS5 and Q methods. On the right, all correlations between 123 leukemia samples are plotted. The samples are from three different array generations U95Av2, U133A, and U133B. The first column illustrates correlations between all replicates together (369 correlation values), and in the other columns the correlations are grouped based on the array generation pairs. When the mean values of the correlations computed with each method were compared, the values in the QAGC data were significantly higher.

We validated the AGC method as well as the entire normalization procedure by a number of ways and demonstrated that we had achieved improved comparability of the data across the multiple array generations. First, analysis by multi-dimensional scaling (MDS) showed that samples from 15 normal human tissues tested clustered initially based on the four array generations (Figure 1a, b), but after the AGC procedure, the tissue of origin was the primary driver of the clustering (Figure 1c, d). Second, in K-means clustering of the same data, we showed that the corrected rand index [23] (a measure of the accuracy of the sample segregation into characteristic clusters) for array generations decreased from 0.45 to 0.15 and that of the tissues jumped from 0.22 to 0.92 (Additional data file 5). Third, correlation of data from two large datasets where the same samples had been analyzed on two different array generations improved significantly after the AGC correction (Figure 2), reaching across-generation correlations of 0.9. Finally, and most importantly, we showed that the gene profiles of multiple previously known tissue-specific genes matched exactly with those expected based on literature data. Therefore, we expect poorly known genes to provide similarly informative results on their biological and medical importance. These various validation steps are described in more detail below.

#### Multi-dimensional scaling analysis

We applied MDS [24] to the data processed by Q normalization alone or after the AGC correction. This was done to compare the variability (that is, noise) caused by the array generation with the biological variability in the data. We evaluated 1,137 healthy tissue samples having 7,390 genes in common without any missing values. The samples represented 15 distinct anatomical locations with more than 20 samples from each site. The samples were measured with four array generations (HG-U133A Plus 2, HG-U133A, HG-U95A and HG-U95Av2). In Q normalized data, only some tissue-associated variation could be observed (Figure 1a), while the clusters were primarily driven by the array generations (Figure 1b). After the AGC step was applied, a major change in the clustering of the samples was seen. Array generations no longer defined clusters (Figure 1c), which were now formed predominantly by the tissue types (Figure 1d). The effect was very striking and defined, for example, a clear cluster of neuronal, muscle, hematological and lung tissues. Even though the MDS in three dimensions gives an illustrative example of the segregation of these 15 tissues types, we do not expect the clusters to be completely separated with MDS and only three dimensions. The main reason is that there is significant biological similarity as well as biological variability within each tissue type (such as multiple overlapping cell types). However, this analysis was not meant to provide a demonstration of complete classification accuracy of human tissues but rather to validate the biological relevance of our data. Taken together, the analysis indicates clear improvement in overall biological relevance of the data after our three-step normalization procedure.

#### K-means clustering

We clustered the data before and after normalization with four initial centroids using the median values of each array generation, and again with 15 initial centroids using the median values of each tissue type. This test was done for the specific purpose of comparing the impact of the variation generated by the array generations before and after normalization. We calculated the corrected rand indices [23] for each clustering to see whether the array generations or the tissue types form more accurate clusters. The corrected rand index compares partitions defined by the K-means clustering to the known partitions of the data (for example, partitions by array generation or by tissue type). The index varies between [1, 0] where one indicates that the partitions are identical and not due to chance, whereas zero indicates that the found partitions would be expected by chance. The corrected rand index for the array generations went down from 0.45 to 0.15 when we applied the AGC normalization, while the corrected rand index for tissues jumped from 0.22 to 0.92. The percentages of samples per array generation and per tissue type segregated to the distinct clusters are given in Additional data file 5.

We also tested the impact of the Q normalization step by performing the same clustering operations on AGC corrected MAS5 data. In this case, the corrected rand index for array generations was 0.11 and for tissue types 0.84. This result showed that AGC could also significantly improve MAS5 data even without the Q normalization, but that the three consecutive steps provided the optimal ability to distinguish biologically relevant signals.

#### Correlations of technical replicates

We then studied the correlations between technical replicates of the same samples analyzed on different Affymetrix array generations. While in itself this does not ensure optimal normalization, such analyses have often been used to compare data from different array generations in previous publications [4,9,25]. Thus, we used data from three datasets as a basis for these analyses [9,26,27]. We first used data for 14 samples of human muscle biopsy samples from patients with inflammatory myopathies [9]. For these cases, data from hybridizations on both HG-U95Av2 and HG-U133A human arrays were available. The correlation coefficient of each replicate pair was > 0.9 when normalized with the AGC method compared to the correlation of the preprocessed and Q normalized values, which were less than 0.75, a significant difference (Figure 2a). We then utilized a dataset from St Jude Children's Research Hospital [26,27] of 123 human leukemias, each analyzed with the three array generations; HG-U95Av2, HG-U133A and HG-U1331B. The mean value of the correlations computed based on the AGC corrected data was significantly higher, 0.78, than the mean of correlations computed based on pre-processed or Q normalized values, which was 0.5 (Figure 2b). For most comparisons, the Q normalized correlations were also slightly higher than those with pre-processing alone.

In summary, validation of the normalization approach (Figures 1a–d, 2a, b; Additional data file 5) together indicate that, in our three-step data processing procedure, the samples clustered mainly according to array generation, until the last AGC correction is applied. After the last AGC step, the biological origin of the samples, and not the array generation, drove the clustering (Figure 1d). Therefore, our *in silico *transcriptomics data have been integrated across all the array generations to the extent that biological variability caused by the tissue and disease types will exceed the technical noise caused by the array generations. This does not mean that the differences between array generations are non-existent, but they will be smaller than most of the biological differences. The final and most important validation of the method was the demonstration that known tissue-and disease-specific genes generated expected profiles across all tissues and diseases (see examples below), thus validating that technical variation is diminished enough to allow accurate biological findings to be made.

### Validating GeneSapiens expression profiles with known tissue-specific genes

**Figure 3:**
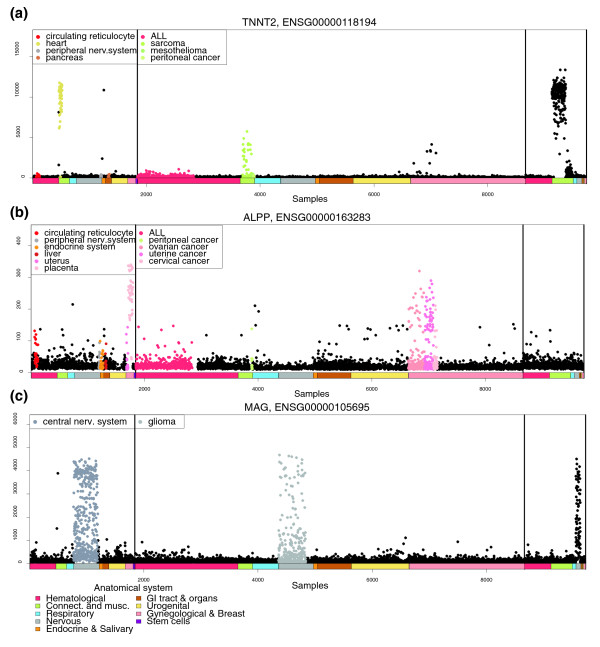
Detailed expression profiles of *TNNT2*, *ALPP *and *MAG*. **(a) ***TNNT2 *is a clinically used cardiac biomarker and, as expected, it shows heart-specific expression. In addition, it has been shown that *TNNT2 *has elevated expression in some cases of rhabdomyosarcoma, also visible from the profile. **(b) ***ALPP *had high expression in placenta and somewhat elevated expression in uterine tumors. Additionally, serous ovarian tumors showed elevated expression when compared to the mucinous ones. **(c) **Known neuronal marker gene *MAG *similarly shows an expression profile that was highly central nervous system specific.

To evaluate the biological relevance of gene expression profiles from *in silico *transcriptomics data, we generated tissue- and disease-wide expression profiles for well-known tissue-specific marker genes. Figure 3 provides examples of the GeneSapiens plots for *TNNT2*, *ALPP *and *MAG*. In these plots, all the 9,783 samples are represented along the x-axis in a pre-determined fixed order, first the normal tissues, then cancers and then other diseases. The y-axis reflects the relative level of gene expression after the three-step normalization approach.

Troponin T (*TNNT2*) showed highly specific expression in heart tissue, as expected for a clinically used cardiac biomarker [28] (Figure 3a). Heart samples in our database originate from four different array generations and comprise only 0.5% of the samples. Therefore, finding an expected tissue-specific expression profile for these samples demonstrates the performance of the normalization even for such a small proportion of samples measured on multiple array generations. Interestingly, *TTNT2 *is also rather highly expressed in many rhabdomyosarcomas and some Muellerian ovarian tumors. There is one report in the literature for a single case of rhabdomyosarcoma showing increased Troponin T levels in serum [29], while our GeneSapiens profile demonstrated that this gene is indeed likely to be upregulated in the two aforementioned tumor types. This demonstrates how GeneSapiens profiles can give additional information even from well-known genes. Expression of placental alkaline phosphatase (PLAP; *ALPP*) was seen predominantly in healthy placenta (Figure 3b), as expected [30], but also often in tumors of the uterus and ovary and rarely in some other tumor types. This observation fits well with the known oncodevelopmental nature of PLAP, with ectopic expression being common in various types of cancers, with uterine and ovarian cancers being particularly well defined as PLAP-positive [31,32]. Finally, *MAG*, a neuronal cell marker [33], showed the highest expression in central nervous system, and to a lesser extent in gliomas (Figure 3c), again a GeneSapiens profile that could be expected for this well-known marker gene.

Additional examples are given in Additional data files 4 and 5, and dozens of known tissue-specific genes or biomarkers can be evaluated through the online tool for exploring tissue- and disease-specific gene expression patterns. For example, *KLK3 *(PSA) is the best-known prostate-specific gene [34] and its GeneSapiens expression profile (Additional data file 2) showed expression only in normal and cancerous human prostate tissues. GFAP is a glial fibrillar acidic protein and showed the expected [35] high level of expression in normal and pathological tissues from the central nervous system (Additional data file 2). Insulin shows the expected extremely pancreas-specific expression (Additional data file 6). LDHC, a known germ-cell specific marker [36], showed a strong testis-specific expression profile (Additional data file 6).

**Figure 4:**
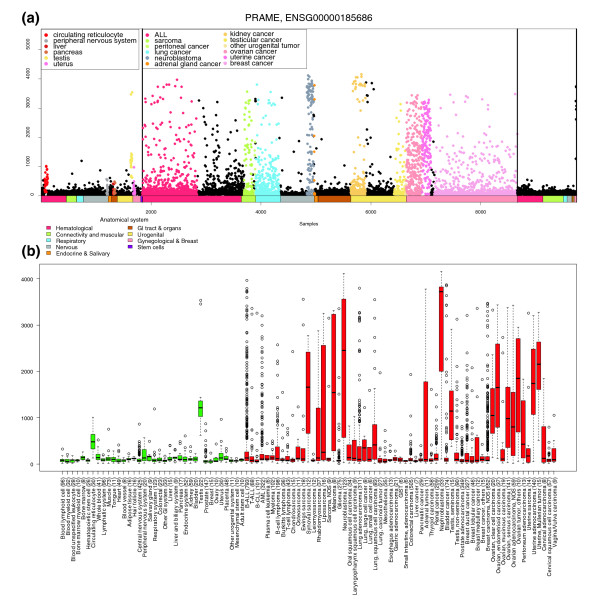
Detailed gene expression profile of *PRAME*. **(a) **Body-wide expression profile of the *PRAME *gene across the database. Each dot represents the expression of *PRAME *in one sample. Anatomical origins of each sample are marked with colored bars below the gene plot. Sample types having higher than average expression or an outlier expression profile are additionally colored in the figure (legend at the top left corner). The *PRAME *gene is a highly testis-specific gene in normal samples, but is ectopically expressed across the majority of human cancers. Gene plots like these can easily be used to identify outlier expression profiles, like as can be seen for kidney cancer in this case, where only a small fraction of the tumors are *PRAME *positive. **(b) **Box plot analysis of the *PRAME *expression levels across a variety of normal and cancer tissues. The number of samples in each category is shown in parentheses. Normal tissues are shown with green boxes and cancerous ones with red boxes. The box refers to the quartile distribution (25-75%) range, with the median shown as a black horizontal line. In addition, the 95% range and individual outlier samples are shown.

GeneSapiens makes it possible to generate gene expression profiles for 17,330 genes across 175 systematically annotated human tissues in a uniform scale with 2,265 to 9,783 data points per gene. Due to the breadth of the tissue and disease spectrum, this kind of analysis provides novel insights into the biological, medical and clinical associations of genes. Furthermore, the expression levels of a given gene can be compared across all normal tissues and all disease types, not just between specific test and control samples (like normal and tumor tissues from the same organ as is usually done). Figure 4a, b illustrate the power of this global tissue- and disease-wide analysis, displaying the expression profile of the *PRAME *gene. *PRAME *(preferentially expressed melanoma antigen) showed high expression in normal testis, but was very highly over-expressed in a large variety of human cancers. *PRAME *over-expression has been previously described in many cancer forms [37] and is known to function as a dominant repressor of retinoic acid receptor signaling [37].

### 'Body-map' analysis to visualize expression profiles for groups of genes across all tissues and diseases

**Figure 5:**
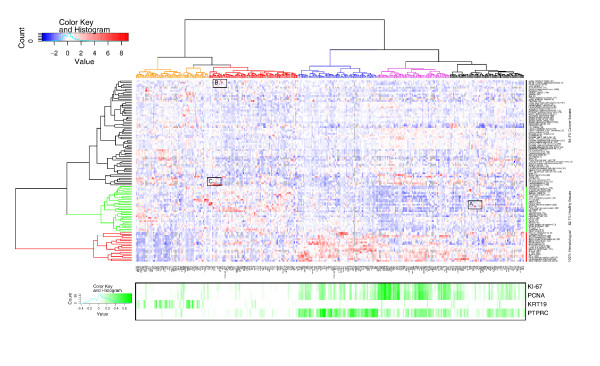
Body-wide expression map of known cancer genes. On the x-axis are 342 genes and on the y-axis are 110 *in vivo *tissues (both healthy and malignant) from human. The color indicates the mean expression value of each gene in each tissue. Grey color signifies missing values. Values have been gene-wise scaled (mean 0 and standard deviation 1). Both axes have been clustered by using Euclidean distance with complete linkage method. Below the expression map are gene-wise Pearson correlation coefficients with four known cellular process/tissue-specific marker genes (*Ki-67*, *PCNA*, *KRT19 *and *PTPRC*). Correlations have been calculated over 8,409 healthy and malignant samples using pairwise complete observations. Comparison of highest correlation values and clusters of genes on the expression map confirm that through the analysis of *in silico *transcriptomics data it is possible to find both tissue specificity and functional associations with processes such as cell cycle. For example, the orange colored branch contains genes having highest correlation with epithelial marker *KRT19*, branches colored blue contain genes mostly expressed in the hematological system and they also correlate with *PTPRC*, a marker for hematological tissues. Additionally, genes related to mitosis cluster together (purple branch), having highest correlations with *Ki-67 *and *PCNA*. The rectangles (A, B, C) highlight three genes as examples of extreme expression in some cancers (see Figure 6 and Additional data files 7 and 8 for enlargements of these areas).

To illustrate the power of GeneSapiens analysis in the study of gene expression profiles of human cancer genes (as defined by Sanger Center human cancer gene census), we produced a clustered map of the mean expression levels of 342 cancer genes across 110 healthy and malignant human tissues (Figure 5). Clustering along the sample type (y-axis) revealed that based on the expression profiles of these cancer genes, the samples could be divided into three overall classes: solid tumors (84.4% of sample types were malignant in this class), normal tissues (82.1% of sample types were healthy in this class) and hematological samples (100% sample types were normal or malignant hematological samples in this class). Thus, the group of classic cancer genes had distinctly different expression between healthy and malignant solid tissues, but in hematological samples, cancer and normal samples could not be separated.

Clustering of the cancer genes according to their mean body-wide expression profiles revealed five characteristic subgroups. Expression of *MKI67 *(Ki-67) [38] and *PCNA *[39] genes, two cell proliferation markers, showed the highest correlations with specific branches of the cancer genes (Figure 5, purple branch). *KRT19 *(a known epithelial marker) [40] and *PTPRC *(an established marker for hematopoiesis) [41] revealed a correlation with genes in the orange and blue branches. Genes most highly associated with proliferation markers were clearly the ones with gain of expression in solid malignant tissues. The branch colored red contained enrichments of Gene Ontology classes [42,43] related to differentiation, cell adhesion and catabolic processes (data not shown), which fits with the tendency for down-regulation of this group of cancer genes in malignant tumors.

**Figure 6:**
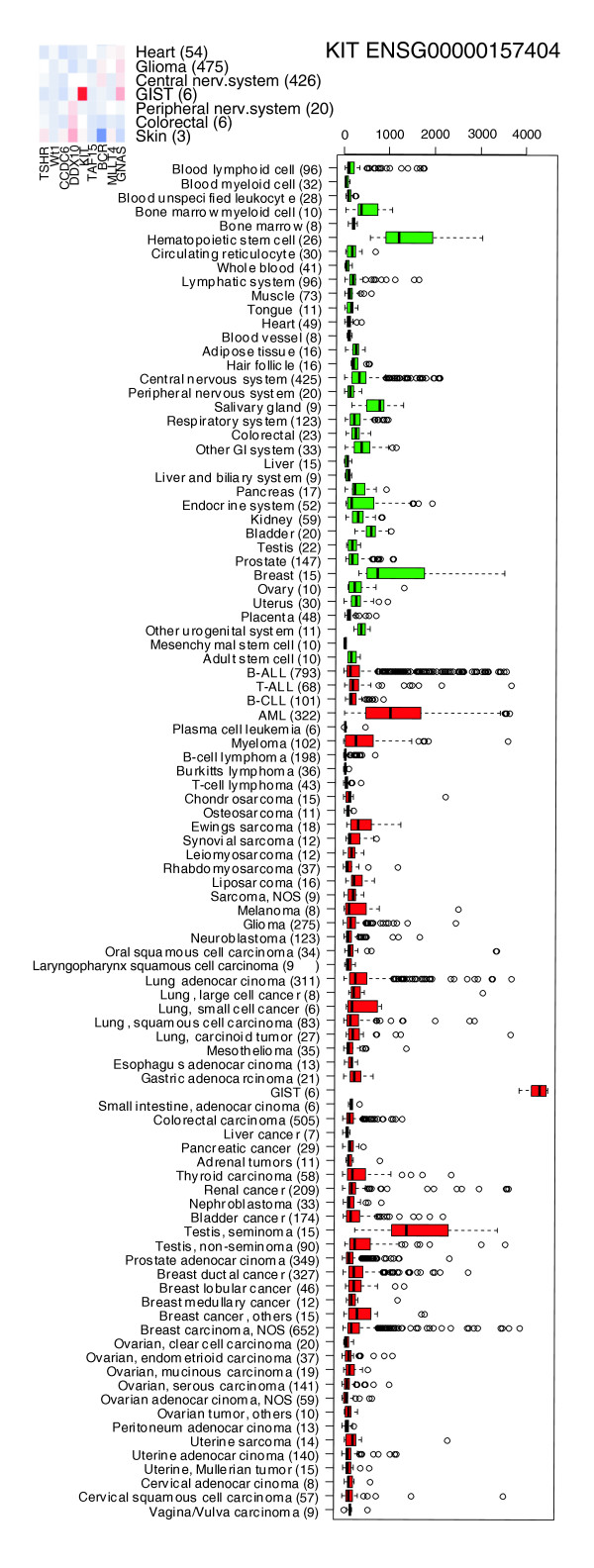
Expression profile for the *KIT *gene shows interesting patterns in the bodymap in Figure 5. *KIT *exhibits extremely high expression in gastrointestinal stromal tumors. *KIT *is known to be inhibited by Gleevec^®^, demonstrating that findings like these pinpoint immediate possibilities for drug repositioning.

This kind of body-wide expression map of genes can also be used to pinpoint medically interesting associations for individual genes (three examples marked with rectangles and labeled A, B and C). *KIT *had the highest GeneSapiens expression level in gastrointestinal stromal tumors (GISTs; Figure 5, rectangle A, and Figure 6). *KIT *is a key therapeutic target of Gleevec in GIST tumors [44]. The body-wide expression profiles of GeneSapiens would have therefore readily identified this association of *KIT *with GIST samples along with this therapeutic opportunity.

The second example is *FEV *(Figure 5, rectangle B, and Additional data file 7), a gene known to have functions in healthy nervous system. This ETS-family transcription factor showed low, but detectable, expression in healthy central nervous system and in prostate. In malignant tissues *FEV *had highly elevated expression in synovial sarcoma, neuroblastoma, malignant peripheral nerve sheath tumors, and small intestinal adenocarcinoma, and somewhat elevated expression in prostate cancer.

The third example of cancer gene profiles is *C1orf56*, also known as *AF1Q *or *MLLT11 *(Figure 5, rectangle C, and Additional data file 8). In healthy tissues it was expressed only in the nervous system, but in malignant tissues there was gain of expression in T-cell acute lymphoid leukemia, Ewing sarcoma, lung small cell cancer, and nephroblastoma, and extreme overexpression in neuroblastoma. *MLLT11 *is known to be fused to the *MLL *gene in acute leukemias [45]. This raises the possibility that *MLLT11 *could be a fusion gene target [46,47] or undergoing activating mutations in a range of tumor types. Alternatively, the high levels of expression in these tumors suggest that this gene is often activated in cancer by other mechanisms.

## Conclusion

The major advantage of the GeneSapiens data mining methodology is that it provides an integrated view of human gene expression levels across thousands of samples representing hundreds of different tissue and disease types. GeneSapiens offers unprecedented possibilities to study gene expression levels not only between a particular tumor type and the corresponding normal tissue, but by providing body-wide overviews of gene expression levels across all kinds of normal and disease states. While meta-analysis of microarray data [48,49] has been previously demonstrated to be powerful in taking advantage of the enormous amounts of publicly available data [1,2,50] most existing methods, such as Oncomine [2] and Genvestigator [51], are based on the analysis of one study at a time. Others, like the Celsius resource, provide the analysis option on one Affymetrix array generation only, therefore providing data from a more limited spectrum of tissues and diseases. In comparison, GeneSapiens provides insights on 'body- and disease-wide' expression of 17,330 genes in approximately 10,000 human samples. Its value is evidenced by the capturing of much of the known data on biological and medical associations for several tissue-specific marker genes (Figures 3, 4, 5, 6), as well as in providing new insights on even well-studied cancer genes. GeneSapiens is characterized by detailed anatomical, histopathological and clinical annotations of disease states, a critically important feature that is often missing in other more generic gene expression database projects.

Virtually every gene we have studied in GeneSapiens has had a distinct pattern of expression across the thousands of samples. Hence, GeneSapiens provides systematic biological and medical annotation of individual human genes, which could prove useful even in the case of relatively well-known and abundantly studied cancer genes. For example, the fact that by far the highest levels of *KIT *expression across all samples available were seen in GISTs demonstrates that one could identify key driver genes that are mutated or otherwise activated in human cancers and could, therefore, be of significant therapeutic significance. This high level of overexpression of *KIT *in GISTs probably reflects the selection pressure favoring the expression of this gene during clonal cancer evolution. GeneSapiens provides the exciting possibility that one could find other previously unknown cancer genes with a similar profile of high expression in one or a few cancer types only that could also turn out to be driven by mutations or translocations [47]. Conversely, even though we will see more and more mutational data being generated from selected human cancers, understanding the impact of the mutations on gene expression will be important. Furthermore, it is extremely useful to be able to characterize the expression of these 'cancer genes' across thousands of cancers and normal tissues of different origins, as sequencing is typically done from a highly selected group of samples. This is illustrated by our analysis of the expression profiles for *FEV *and *C1orf56 *(*MLLT11)*. Besides the therapeutic importance, the data on several serum biomarkers of disease, such as Troponin T and PSA, indicate that the body-wide expression profiles of genes could highlight genes with a high specificity to a single organ or disease type, and, therefore, with potential value as serum biomarkers.

The third important aspect of the GeneSapiens system is the interactive nature of the analysis options that we have generated for making these data publicly available in a user-friendly format. We have set up an interactive website [16] to provide access to the *in silico *transcriptomics data with detailed expression profiles for 17,330 genes across all the 9,783 annotated healthy and pathological human samples. We provide the possibility to analyze the levels of gene expression across all the tissues and malignant diseases (box-and-whisker plots; Figure 3a–d), as well as to analyze gene expression at the level of individual samples. The 'GeneSapiens plot' (see, for example, Figure 4a) displays expression levels of the genes in each of the 10,000 samples, arranged in anatomical order and by disease type. The datapoints displayed are interactive and provide links to the specific type of the sample, the histopathological diagnosis and the type of the array generation used. We also provide filtered analysis options where users can explore in detail a particular organ or disease type as well as the option of analyzing the correlation of any two genes across the whole database or subsets of tissues or diseases. Taken together, we believe that the GeneSapiens analysis system provides a highly useful resource to the biomedical research community.

## Materials and methods

### Data collection

This *in silico *collection of human transcriptomes was constructed by collecting 9,783 publicly available Affymetrix microarray experiments in the form of CEL files as source material. The uniqueness of the collected files was tested with the cyclic redundancy check algorithm (cksum). For a complete listing of the original source data from 157 separate studies, please see Additional data file 3. We combined data from the following Affymetrix generations (HG-U95A, HG-U95Av2, HG-U133A, HG-U133B, HG-U133 Plus 2). Even though HG-U133A and HG-U133B are not different generations, they do have 2,074 common genes, and we considered them as such for the practical purposes of our normalization.

### Data preprocessing

Data from all CEL files were pre-processed with the MAS5.0 algorithm [18] with default parameters. Although different opinions exist about optimal preprocessing methods [52], recent comparison studies indicate that MAS5.0 provides the most faithful cellular network construction [53] and optimal identification of differentially expressed genes [54]. In addition, other preprocessing methods may create false positive results [53,55,56]. We used version 10 of the alternative CDF files [20] summarizing the probe level intensities directly to the Ensemble [57] gene IDs (Ensembl build 46). Probes mapping to multiple genes and other problems associated with old generations of Affymetrix probe designs were thereby excluded. Within our normalization process the term pre-processing refers only to steps performed by the MAS5.0 algorithm, and subsequent normalization steps are described below.

### Sample-wise normalization with equalization transformation

We utilized equalization transformation (Q) [21], a method similar to widely used quantile normalization [22], to normalize the pre-processed data. After Q normalization, the dataset had the desired distribution that has been determined prior to transformation. The normal distribution with mean of 8 and standard deviation 2 (*N*(*8, 4*)) was selected as the desired distribution since the distribution of logarithmic, preprocessed values of all samples (N = 9,783) with median 7.92 and standard deviation 2.3 was near to this distribution (Additional data file 1). EQ values were brought to exponential scale to maintain the scale of the original values.

The quantile normalization [22] would be another choice to perform normalization but has considerable drawbacks in this particular setting. First, it does not perform well when there is variation in the number of genes between samples. This problem is magnified when merging thousands of samples from different array generations. Also, the means of the quantiles may vary substantially when new samples are added to the dataset, whereas the change caused by the equalization transformation is smaller. Quantile normalization is also resource-intensive to compute for thousands of samples with different numbers of measured genes. Thus, equalization transformation (Q) [21] was the method of choice in this study.

### Array-generation-based gene centering (AGC)

To be able to compare the samples of *in silico *transcriptomics also between the array generations, we developed a novel method for gene-wise normalization of the data. In this AGC method we assume that the mean of the expression values for any particular gene in each array generation is the same. If the mean value of some of the array generations differs substantially from the others, the shift is assumed to be caused by the array generation based variation, and the AGC method aims to correct this variation. The AGC method requires that the collection of samples to be analyzed is large enough so that one can assume the distribution of values of each gene *k *to represent the total distribution of all potential expression values across all tissues for each array generation *i*. Therefore, the AGC method normalizes the data to have mean values *μ*_*i, k*_*= μ*_*all, k *_for all array generations *i*, where *μ*_*all, k *_is the mean of all values of the gene *k*. Further, it is assumed that the minimum and the maximum estimates for the gene value are reached and the range of the gene *k *should approximately be [*a*_*k*, _*b*_*k*_], where *a*_*k *_is the lowest 2% value and *b*_*k *_is the largest 2% value of gene *k*. AGC values should not go over this range. However, if the new centered value exceeds the range, the difference is diminished towards the range limits with coefficient *c*, 0 ≤ *c *≤ 1. Here, the coefficient is set to *c *= 1/5. Coefficient *c *is necessary to prevent some extremely tissue-specific genes from having arbitrarily large correction factors, which is possible if the specific tissue is absent from one or more array generation. The coefficient *c *affects 2.9% of all correction factors. Of those cases, the proportion of the correction factor modified by coefficient *c *was, on the average, 7.6%. Thus, the coefficient *c *affected an extreme minority of the corrections in a significant manner, but nevertheless, it was found to be crucial for the AGC method. The centered values can now be obtained with:

$${\hat{x}}_{i,j,k}=x_{i,j,k}-(\mu_{i,k}-\mu_{all,k})$$

where *x*_*i*, *j*, *k *_is the value of gene *k *in sample *j *from array generation *i*, *μ*_*i*, *k *_is the mean of the values of gene *k *across array generation *I*, and *μ*_*all*, *k *_is the mean of the values of gene *k *across all array generations. Further, the adjusted values are computed based on the equation:

$$y_{i,j,k}=\{\begin{array}{ll} b_{k}+c({\hat{x}}_{i,j,k}-b_{k}), & \text{for }{\hat{x}}_{i,j,k}>b_{k},\\ a_{k}-c(a_{k}-{\hat{x}}_{i,j,k}), & \text{for }{\hat{x}}_{i,j,k}<a_{k},\\ {\hat{x}}_{i,j,k}, & \text{otherwise}\text{.} \end{array}$$

The resulting AGC values are now AGCvalue = 2^*y*^.

Some other methods [58,59] are useful to combine different datasets. However, these are computationally very demanding and probably impractical for datasets comprising almost 10,000 samples. Additionally, the performance of these methods is not validated for integration of multiple datasets.

### Sample annotation and manual curation

Annotation of the samples is important to make biological and medical sense of the data. Since not all sources of CEL files come with annotations following the MIAME standards [60], we performed manual annotation of all the data in the database. Annotation terms linked to each sample were defined by a team of seven biologists and medical doctors. The content of the database in terms of healthy, malignant and other disease samples can be seen in Additional data file 4

### Gene annotation

Gene annotation is based on Ensembl. The database has data for each Ensembl gene, even those not featured on any arrays. Gene data include transcript and protein product information, chromosome name and position (band and nucleotide count), biotype (protein coding, miRNA, ribosomal, and so on), and Hugo and Entrez IDs for each gene. These data were downloaded from the Ensembl web site, using the same Ensembl genome build version (release 46) as that used for the construction of the used alternative CDF files [20].

### Multidimensional scaling and clustering accuracy

We utilized classic MDS in order to diminish the number of the dimensions within the data [24]. With MDS, 1,137 samples with 7,390 dimensions (that is, genes) were brought to low-dimensional space so that the distance between each sample pair with these new dimensions is very close to the distance between the original values of the samples. As a distance metric, we used Manhattan distance.

### K-means clustering and rand index analysis

K-means clustering was performed with default parameters in R. The initial centroids were given as the median value of each gene in array generations or tissues. The algorithm was allowed to run for a maximum of 100,000 iterations for each clustering. The corrected rand index [23] was calculated in R with fpc library.

### Replicate analysis

Replicate analysis was performed by comparing the correlation coefficients of the logarithmic values of two or three hybridizations from a single biological sample using standard methods of computing the Pearson correlation coefficient. This was done for all samples described in [9,26,27].

### Body-wide expression profiles of genes

We visualize the expression profile of a single gene across all human tissues with boxplots and with custom designed body-wide expression plots. In the boxplots, the expression profiles of a single gene are displayed and grouped into healthy samples (green boxes) and malignant samples (red boxes). Both types are in anatomically meaningful order, allowing easy comparison of related tissue types. Numbers of samples in each tissue type are in parentheses.

Custom designed body-wide expression profiles show the expression pattern of a single gene at the level of individual samples, while its layout allows easy analysis of the biological or medical significance of the profile. The y-axis provides the expression level of the gene and the x-axis contains all samples arranged into a fixed order by the type of the sample (healthy, malignant) and subsequently by the tissue type. Thus, each dot describes the expression level of a particular gene in one sample. The anatomical origin of each sample can be seen from the color bar at the bottom of the image. Tissues expressing the gene at a high level (more than one standard deviation higher than the baseline for that gene or having a group of outlier data points) are colored.

### Body-wide gene expression heatmaps for human cancer genes

Bodywide expression maps of genes are done with hierarchical clustering (Euclidean distance with Ward linkage) of mean expression profile for 342 genes across 110 *in vivo *tissues. The number of samples per tissue type is given in parentheses. Values for each gene are mean-centered at 0 with a standard deviation of 1.

### Availability of data

As the *in silico *transcriptomics data of this project are composed of custom integration of already public microarray data we provide a table describing the origins of the data used to construct GeneSapiens (Additional data file 3). We have set up a website [16] to allow browsing of expression profiles of these genes and associated information as well as generation of correlations/scatterplots between any pairs of genes across any tissues.

## Abbreviations

AGC: array-generation-based gene centering; GIST: gastrointestinal stromal tumor; MDS: multi-dimensional scaling; PLAP: placental alkaline phosphatase; Q: quantile; QAGC: Q normalized data to which AGC correction has been applied.

## Competing interests

The institute has filed a patent application regarding the normalization methodology.

## Authors' contributions

SK contributed to the majority of data analysis, database construction and development of normalization and writing of the manuscript. RA and MS contributed to the development and testing of the normalization. KO and EB contributed to data collection and the annotation process. KO also contributed to data mining methods and checking of all annotations. KI had a major contribution to the annotation. SH contributed to the development of normalization and supervised the comparison and validation of the normalization methods. OK supervised the entire project for database construction, data mining and annotation efforts and participated in manuscript writing and editing. The remaining authors contributed towards annotation, data visualization and other methods as well as editing the manuscript.

## Additional data files

The following additional data are available with the online version of this paper. Additional data file 1 shows the distribution of preprocessed datapoints across the entire database (solid line) and normal distribution (*N*(*8, 4*)) estimated from it (dashed line). Additional data file 2 shows boxplots of various known tissue-specific genes. Additional data file 3 lists the sources for all the raw expression data files used in this study. Additional data file 4 lists the various healthy tissues, cancers and non-cancer diseases represented by the samples in the database and the amounts of samples in each of these categories. Additional data file 5 lists rand indices for the different normalizations, and the distribution of array generations and tissues into clusters with Q and QAGC (Q normalized data to which AGC correction has been applied) normalized data. Additional data file 6 shows boxplots of various known tissue-specific genes. Additional data file 7 shows that *FEV *has clearly elevated expression in several malignancies, when compared to any healthy tissue. Most interestingly this ETS-factor family member appears to have slightly elevated expression in prostate cancer when compared to healthy prostate. Additional data file 8 shows that expression of the *C1orf56 *gene, also known as *AF1Q *or *MLLT11*, shows extreme expression in several cancers, especially in neuroblastoma.

## Supplementary Material

Additional data file 1 Distribution of preprocessed datapoints across the entire database (solid line) and normal distribution (*N*(*8, 4*)) estimated from it (dashed line). Click here for file

Additional data file 2 **(a) ***KLK3 *(PSA) is a known prostate specific gene. This specificity is perfectly shown in its expression profile. **(b) ***GFAP*, a gene coding for glial fibrillary acidic protein, is known to be expressed in central nervous system. Its expression profile perfectly confirms this prior knowledge. Click here for file

Additional data file 3 Sources for all the raw expression data files used in this study. Click here for file

Additional data file 4 Various healthy tissues, cancers and non-cancer diseases represented by the samples in the database and the amounts of samples in each of these categories. Click here for file

Additional data file 5 Rand indices for the different normalizations, and the distribution of array generations and tissues into clusters with Q and QAGC normalized data. Click here for file

Additional data file 6 **(a) **Insulin (INS) has pancreas specific expression, as one expects it to have. **(b) ***LDHC *is a known testis-specific gene and it is expressed above background only in healthy testis. Click here for file

Additional data file 7 Most interestingly this ETS-factor family member appears to have slightly elevated expression in prostate cancer when compared to healthy prostate. Click here for file

Additional data file 8 Expression of the *C1orf56 *gene, also known as *AF1Q *or *MLLT11*, shows extreme expression in several cancers, especially in neuroblastoma. Click here for file
