# Supplementary figures and images for: Systematic bioinformatic analysis of expression levels of 17,330 human genes across 9,783 samples from 175 types of healthy and pathological tissues
Source: Genome Biol. 2008 Sep 19;9(9):R139. doi: 10.1186/gb-2008-9-9-r139 (PMC2592717; doi:10.1186/gb-2008-9-9-r139)

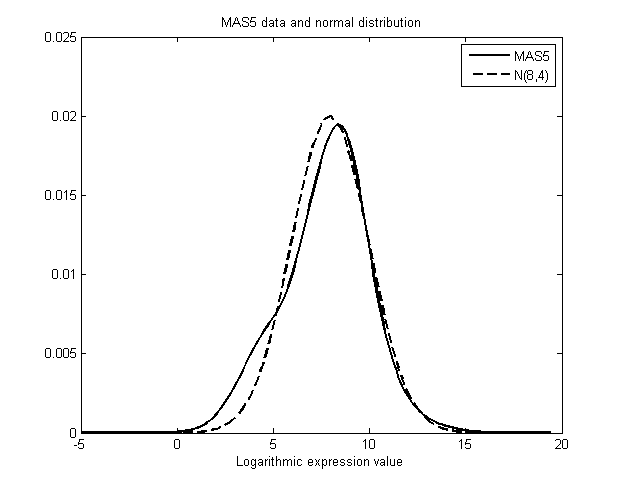

Supplement: Additional data file 1 — Distribution of preprocessed datapoints across the entire database (solid line) and normal distribution (N(8, 4)) estimated from it (dashed line). [file gb-2008-9-9-r139-S1.png]

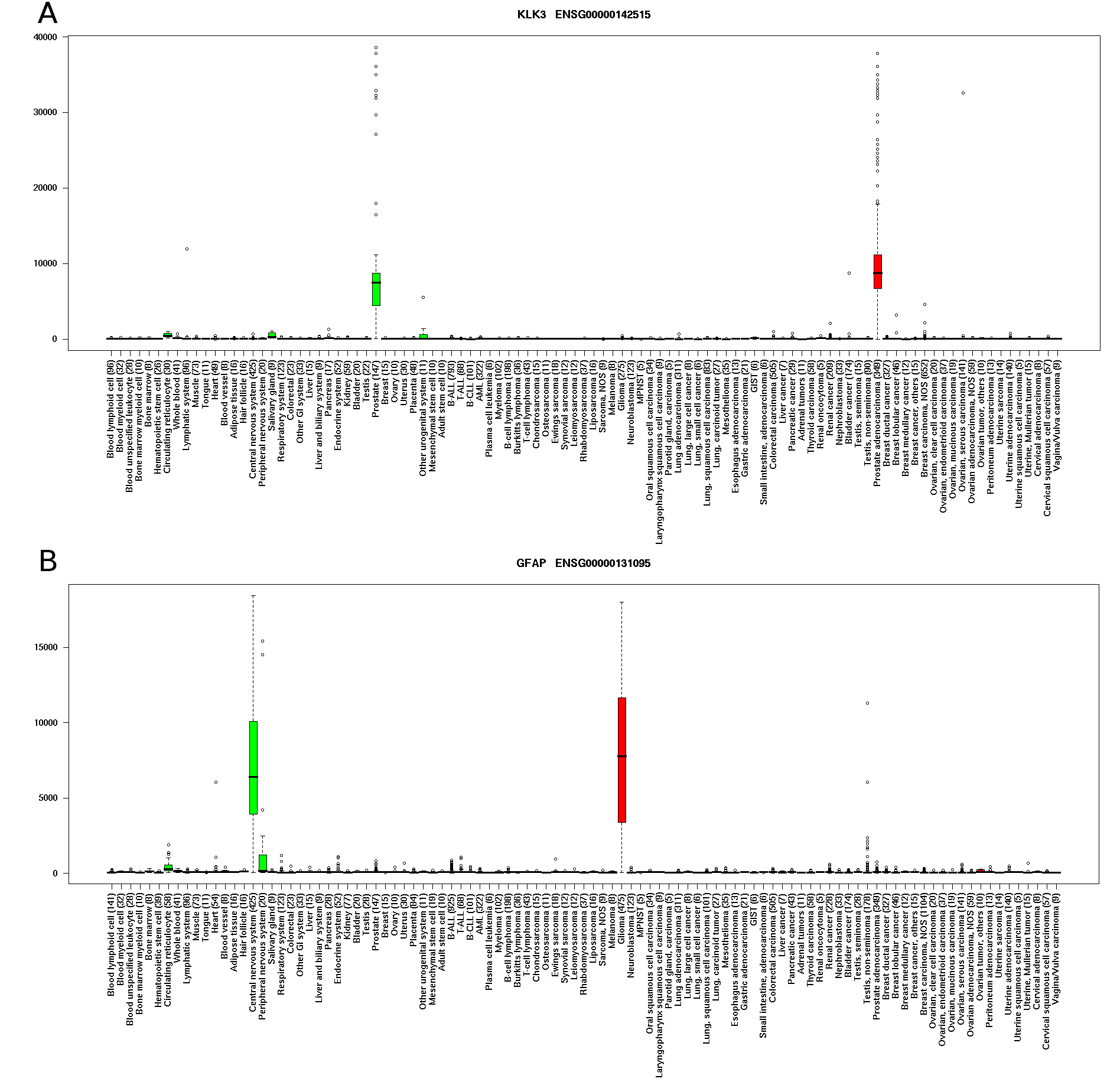

Supplement: Additional data file 2 — (a) KLK3 (PSA) is a known prostate specific gene. This specificity is perfectly shown in its expression profile. (b) GFAP, a gene coding for glial fibrillary acidic protein, is known to be expressed in central nervous system. Its expression profile perfectly confirms this prior knowledge. [file gb-2008-9-9-r139-S2.png]

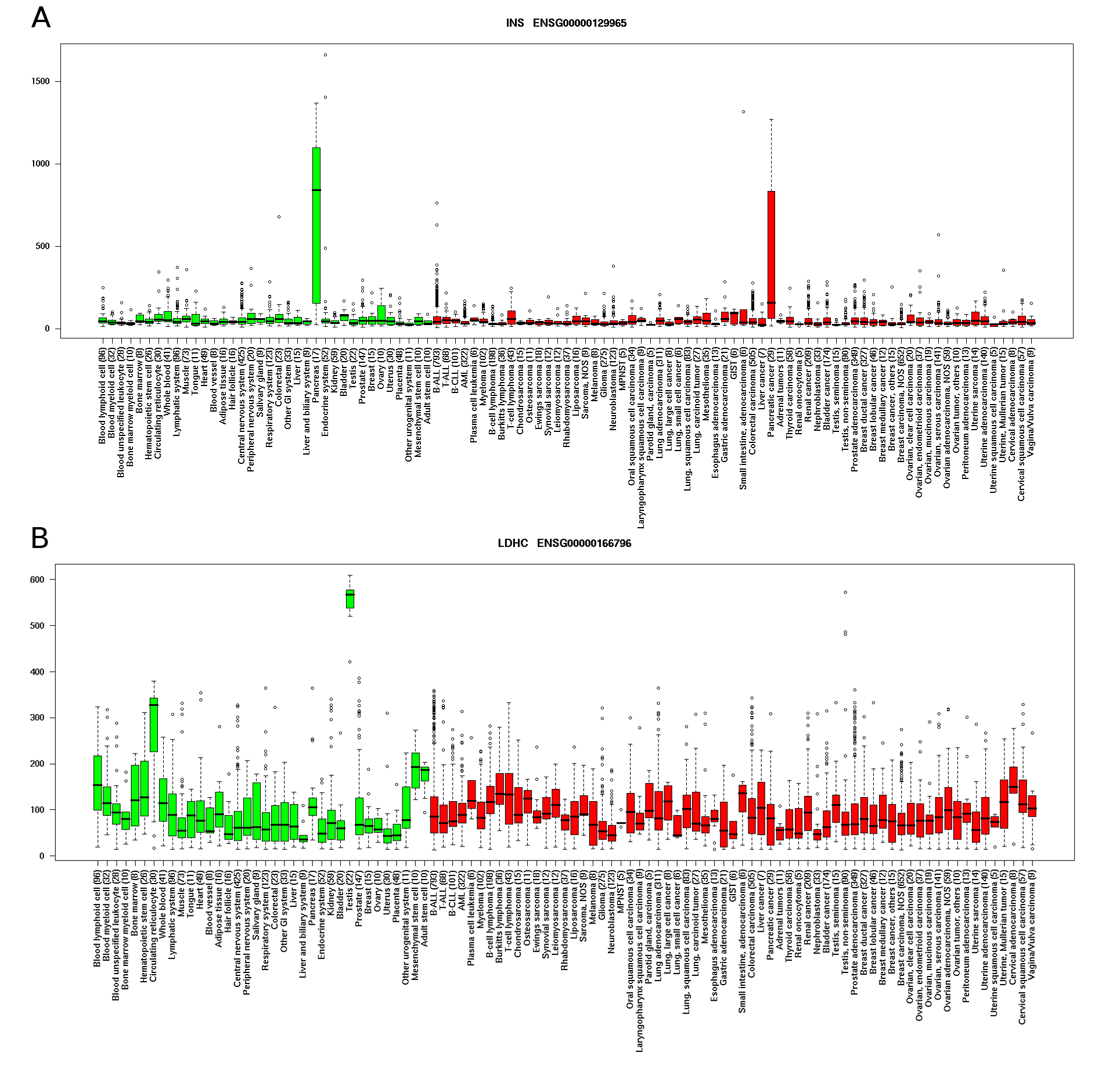

Supplement: Additional data file 6 — (a) Insulin (INS) has pancreas specific expression, as one expects it to have. (b) LDHC is a known testis-specific gene and it is expressed above background only in healthy testis. [file gb-2008-9-9-r139-S6.png]
